# Randomized Clinical Trial Comparing On-clamp Versus Off-clamp Laparoscopic Partial Nephrectomy for Small Renal Masses (CLOCK II Laparoscopic Study): A Intention-to-treat Analysis of Perioperative Outcomes

**DOI:** 10.1016/j.euros.2022.10.007

**Published:** 2022-10-28

**Authors:** Riccardo Bertolo, Pierluigi Bove, Marco Sandri, Antonio Celia, Luca Cindolo, Chiara Cipriani, Mario Falsaperla, Costantino Leonardo, Andrea Mari, Paolo Parma, Alessandro Veccia, Domenico Veneziano, Andrea Minervini, Alessandro Antonelli, AGILE Group (Italian Group for Advanced Laparoendoscopic Surgery)

**Affiliations:** a Urology Department, San Carlo di Nancy Hospital, Rome, Italy; b Urology Unit, Department of Surgery, Tor Vergata University of Rome, Rome, Italy; c Data Methods and System Statistical Laboratory, University of Brescia, Brescia, Italy; d Department of Urology, San Bassiano Hospital, Bassano Del Grappa, Italy; e Department of Urology, Villa Stuart Private Hospital, Rome, Italy; f Department of Urology, ARNAS Garibaldi Hospital, Catania, Italy; g Department of Urology, La Sapienza University of Rome, Rome, Italy; h Department of Urology, Careggi Hospital, University of Florence, Florence, Italy; i Department of Urology, Ospedale “Carlo Poma” Mantova, Mantova, Italy; j Unit of Urology, Spedali Civili Hospital, University of Brescia, Brescia, Italy; k Department of Urology and Kidney Transplantation, O.O. Riuniti BMM, Reggio Calabria, Italy; l Department of Urology, Azienda Ospedaliera Universitaria Integrata of Verona, University of Verona, Verona, Italy

**Keywords:** Partial nephrectomy, Renal neoplasm, Laparoscopy, Off-clamp, Clampless, Ischemia

## Abstract

**Background:**

Recent randomized trials (RCTs) in the field of robotic partial nephrectomy (PN) showed no significant differences in perioperative outcomes between the off- and on-clamp approaches.

**Objective:**

To compare the perioperative outcomes of on- versus off-clamp pure laparoscopic PN (LPN).

**Design, setting, and participants:**

A multi-institutional analysis of the on- versus off-clamp approach during LPN in the setting of an RCT (CLOCK II trial; ClinicalTrials.gov NCT02287987) was performed.

**Intervention:**

Off- versus on-clamp LPN.

**Outcome measurements and statistical analysis:**

Baseline patient and tumor variables, and peri- and postoperative data were collected. Randomized allocation with a 1:1 ratio was assigned. Surgical strategy for managing the renal pedicle was dictated by the study protocol. In the off-clamp arm, the renal artery had to remain unclamped for the duration of the whole procedure. Reporting the intention-to-treat analysis is the purpose of the study.

**Results and limitations:**

The study recruited 249 patients. Of them, 123 and 126 were randomized and allocated into the on- and off-clamp treatment groups, respectively. Treatment groups were comparable at baseline after randomization with respect to patients’ demographics, comorbidities, renal function, and tumor size and complexity. A univariable analysis found no differences in the perioperative outcomes between the groups, including median (interquartile range) estimated blood loss (150 [100–200] vs 150 [100–250] ml, *p* = 0.2), grade ≥2 complication rate as classified according to the Clavien-Dindo system (5.7% vs 4.8%, *p* = 0.6), and positive surgical margin rate (8.2% vs 3.5% for the on- vs off-clamp group, *p* = 0.1). No differences were found in terms of the 1st (81.3 [66.7–94.3] vs 85.3 [71.0–97.7] ml/min, *p* = 0.2) and 5th postoperative days estimated glomerular filtration rate (83.3 [70.5–93.7] vs 83.4 [68.6–139.3] ml/min, *p* = 0.2). A multivariable analysis found each +1 increase in RENAL score corresponded to an increase in the protection from the occurrence of complications (odds ratio [OR] 0.72, 95% confidence interval [CI] 0.54–0.97, *p* = 0.034), while each +1 cm increase in tumor size corresponded to an increase in the risk of blood transfusion (OR 1.39, 95% CI 1.14–1.70, *p* = 0.001).

**Conclusions:**

In the setting of an RCT, no differences were found in the perioperative and early functional outcomes between on- and off-clamp LPN.

**Patient summary:**

In this study, we investigated, by means of a randomized trial, whether avoiding the clamping of renal artery during laparoscopic resection of renal mass is able to translate into benefits. We found no differences in terms of safety, efficacy, and renal function from the standard approach, which includes arterial clamping.

## Take Home Message

In the setting of a randomized controlled trial, no differences were found in the perioperative and early functional outcomes between on- and off-clamp laparoscopic partial nephrectomy.

## 1 Introduction

The current guidelines recommend partial nephrectomy (PN) whenever feasible as a surgical treatment option for T1 renal masses suspicious for cancer [1]. Together with the complete removal of the mass (the “leave no tumor behind” concept [2]), PN has gained broader diffusion over radical nephrectomy with the ideal goal of preserving more renal function [3]. Several factors interplay in the residual renal function after PN [4]: patient-related variables, including age, comorbidities, and baseline kidney function, represent unmodifiable factors, while surgery-related variables, such as the technique for managing the renal pedicle, resecting the tumor, and suturing the renal defect, are modifiable factors [5].

Since the beginning of the “PN saga,” the ischemic injury deriving from long ischemia intervals during the clamping of renal artery has been named among the more detrimental factors responsible for the postoperative renal function decrease [6], [7]. As such, it has been hypothesized that choosing an off-clamp approach might better preserve the ultimate renal function. If during open PN an “off-clamp” technique is often performed, given the direct hand-assisted control over the renal pedicle allowed to the surgeon [8], during laparoscopic PN (LPN), the clamping of renal artery has been considered crucial in order to obtain a bloodless field during the tumor excision. Challenges related to the pure laparoscopic approach increase the risk of longer ischemia times, particularly in less experienced hands. This is why the clamping of the main renal artery has been challenged by several “alternative” techniques aimed to shorten/avoid renal ischemia. The exasperation of the concept has led to the “no ischemia” approach first described by Guillonneau et al. [9] and duplicated by other authors [10], [11]. Off-clamp LPN is a technically demanding approach, requiring consistent experience before embarking on it. Some authors questioned off-clamp LPN as potentially negatively impacting the perioperative outcomes of the surgery, increasing blood loss, worsening the vision of the operative field, and sponsoring the likelihood of complications and positive surgical margins.

Actually, a recent systematic literature review including pure LPN showed no impact on either surgical or oncological outcomes by the off-clamp technique [12], but the finding is debatable and based on the pooled analysis of mostly small sample size studies, affected by several confounders, including a selection bias (the off-clamp approach is more likely to be performed in low-complexity renal masses by more expert surgeons).

Our research group sought to contribute to the field with the aim of increasing the level of evidence about the topic. A randomized clinical trial (RCT), the “CLOCK II” study (CLamp vs Off Clamp of the Kidney during laparoscopic partial nephrectomy; ClinicalTrials.gov NCT 02287987) was conceived. The purpose of the study was to compare the perioperative outcomes of on- versus off-clamp LPN.

## 2 Patients and methods

### 2.1 Study protocol

The CLOCK II laparoscopic study is a multicentric RCT, promoted by the AGILE group (the Italian group for advanced laparoendoscopic surgery; www.agilegroup.it), which started in January 2015. The study received ethics committee approval (registration number NP 1814). All enrolled patients had been informed and signed a consent form. In this RCT, after accounting for the inclusion/exclusion criteria, consecutive candidates to LPN at six institutions were randomized to on- or off-clamp LPN. A single experienced laparoscopic surgeon with a detailed surgical profile (<45 yr old, prior experience in laparoscopic renal surgery, and at least 100 LPNs with different clamping approaches performed as first operator prior to the study start) was involved per center. The study protocol has already been described fully elsewhere [13]. Briefly, only patients with normal coagulative parameters, preoperative estimated glomerular filtration rate (eGFR) >60 ml/min (estimated by radionuclide renal scan), normal contralateral kidney at baseline renal scan, and complexity of the renal mass ≤10 as assessed by the RENAL score [14], were included. All patients participating in clinical research studies investigating either drugs or therapies administered within 6 mo prior to the enrollment, and/or patients diagnosed with neuropsychiatric abnormalities, and/or patients unable to attend the scheduled follow-up visits were excluded.

### 2.2 Randomization

The random sequence for the two comparison groups (on- vs off-clamp) was computer generated using the command *ralloc* in Stata 15. Randomized allocation with a 1:1 allocation ratio was assigned by a permuted block design, stratified by center [13], [15]. Randomization was also stratified according to the complexity of the tumor based on the RENAL score (<7 vs ≥7). The allocation arm was notified by means of a dedicated e-form, managed by an independent software house. At any moment, the investigators were able to amend the indication given by the randomization and shift the patients to the alternative option for managing the renal artery.

### 2.3 Outcome measurements

Patients’ demographics, baseline patient and tumor variables, and peri- and postoperative data were collected. Specifically, patients’ comorbidities were classified according to the Charlson’s index [16]. The resection technique was classified by the surface-intermediate-base score [17].

As stated, the surgical strategy for managing the renal pedicle was dictated by the study protocol. Perirenal fat and renal artery dissection were mandatory surgical steps in all patients.

Neither preoperative transarterial embolization nor intraoperative controlled hypotension was allowed. When randomized in the on-clamp treatment group, tumor resection and inner renorrhaphy layer had to be done with the renal artery clamped. On the contrary, in the off-clamp arm, the renal artery had to remain unclamped for the whole procedure duration.

Since the start till the conclusion of the tumor resection/parenchymal renorrhaphy, the investigators were able to waive at any moment the indication given by the randomization and shift to the alternative renal artery management option. Details and reasons for deciding for a shift had to be reported mandatorily. As concerning the perioperative outcomes, the severity of the eventual intraoperative bleeding was categorized on a scale from 0 (=no bleeding) to 5 (=intense bleeding exceeding the suction cannula capacity). Postoperative complications were classified according to the Clavien-Dindo system [18].

### 2.4 Statistical analysis

The target sample size has been calculated using the Borm, Fransen, and Lemmens formula for analysis of covariance (ANCOVA). As reported in the published study protocol [15], we assumed from previous reports a standard deviation *σ* of 20 ml/min/1.73 m^2^. For *α* = 5%, 1 − *β* = 80%, a *ρ*^2^ of 0.6, and a clinically significant minimum difference *δ* = 5 ml/min/1.73 m^2^, the minimum required sample size was calculated to be 102 patients per arm. Actually, 113 patients per arm were considered the target enrolment after adjusting for a 10% chance of dropping out.

Reporting the intention-to-treat analysis of the patients’ demographics, baseline characteristics, and perioperative outcomes is the purpose of the study.

Categorical variables were summarized as absolute and relative frequencies. Numerical variables were reported as means and standard deviations or medians and interquartile ranges (IQRs), as appropriate. The comparison of the average eGFR and hemoglobin pre- versus postoperative variations in the treatment groups (off- vs on-clamp LPN) was conducted by using the ANCOVA. The comparison of the median values of the total operative and renorrhaphy times in the study groups was performed by using the nonparametric Mann-Whitney test for independent samples. The complication rates were compared by Fisher's exact test. The association between clinical features and the event of shifting from off- to on-clamp LPN was investigated by using a binary logistic regression and measured by odds ratios (ORs). All tests were two sided. A *p* value of <0.05 was considered significant. Statistical analysis was performed by using Stata 15.0 (Stata Statistical Software: release 15, 2017; StataCorp LLC, College Station, TX, USA).

## 3 Results

As of September 2019, the CLOCK II laparoscopic study recruited 249 patients. Of them, 123 and 126 were randomized and allocated to the on- and off-clamp treatment groups, respectively. The number of enrolled patients exceeded the planned sample size [13]. At baseline, the on- and off-clamp treatment groups were well balanced as concerning the patients’ demographics, coagulative function, comorbidities, renal function, and clinical tumor size and complexity. Complete baseline data are reported in Table 1.

**Table 1:** Baseline variables, patients’ demographics, coagulative function, comorbidities, renal function, and lesion’s characteristics

Variables	On clamp	Off clamp	*p* value
Males, no. (%)	85 (69.1)	88 (69.8)	1
Age (yr), median (IQR)	61.0 (50.5–70.0)	60.0 (52.2–68.8)	0.7
BMI, median (IQR)	26.4 (24.2–29.0)	26.8 (24.4–29.3)	0.5
Platelet count, median (IQR)	232k (186k–278k)	226.5k (188k–270k)	0.7
Hb (g/dl), median (IQR)	14.0 (13.0–14.8)	14.3 (13.4–15.2)	0.03
% Hct, median (IQR)	42.3 (40.1–44.2)	43.0 (41.4–45.0)	0.07
PT (s), median (IQR)	95.0 (88.0–99.0)	95.5 (88.0–99.0)	0.8
PTT (s), median (IQR)	30.0 (28.2–35.8)	29.7 (28.0–35.9)	0.3
Hypertension, no. (%)	62 (50.4)	77 (61.6)	0.1
Diabetes, no. (%)	25 (20.3)	25 (20.3)	1
Cardiac disease, no. (%)	13 (11.1)	14 (11.6)	1
Vasculopathy, no. (%)	15 (12.3)	20 (16.4)	0.5
ECOG performance status, no. (%)			1
0	95 (77.9)	96 (76.8)	
1	24 (19.7)	25 (20)	
≥2	3 (2.5)	4 (3.2)	
Charlson’s Comorbidity Index, no. (%)			0.8
0	62 (51.2)	62 (50)	
1	12 (9.9)	13 (10.5)	
2	11 (9.1)	14 (11.3)	
>2	36 (29.7)	35 (28.2)	
SCr (mg/dl), median (IQR)	0.9 (0.8–1.0)	0.9 (0.8–1.0)	0.6
eGFR (ml/min), median (IQR)	90.0 (71.0–99.0)	90.0 (70.0–99.0)	0.8
% Split renal function, median (IQR)	45.0 (30.0–52.0)	45.0 (39.5–52.2)	0.3
Clinical tumor size (cm), median (IQR)	3.0 (2.6–4.5)	3.0 (2.3–4.1)	0.3
RENAL score, median (IQR)	6.0 (5.0–8.0)	6.0 (5.0–7.0)	0.8

BMI = body mass index; ECOG = Eastern Cooperative Oncology Group; eGFR = estimated glomerular filtration rate; Hb = hemoglobin; Hct = hematocrit; IQR = interquartile range; PT = prothrombin time; PTT = partial thromboplastin time; SCr = serum creatinine.

The median (IQR) eGFR was 90.0 (71.0–99.0) versus 90.0 (70.0–99.0) ml (*p* = 0.8) for the on- versus off-clamp procedure, with equal median % split renal function (45.0 [30.0–52.0] vs 45.0 [39.5–52.2], *p* = 0.3). Both clinical tumor size (3.0 [2.6–4.5] vs 3.0 [2.3–4.1] cm, *p* = 0.3) and tumor complexity as assessed by the RENAL score (6.0 [5.0–8.0] vs 6.0 [5.0–7.0], *p* = 0.8) were comparable between the groups. A univariable analysis found no differences in the perioperative outcomes between the groups, including median (IQR) estimated blood loss (150 [100–200] vs 150 [100–250] ml, on- vs off-clamp, *p* = 0.2), Clavien grade ≥2 complication rate (5.7% vs 4.8%, on- vs off-clamp, *p* = 0.6), and positive surgical margin rate (8.2% vs 3.5%, on- vs off-clamp, *p* = 0.1). Complete results are reported in Table 2.

**Table 2:** Perioperative outcomes

Variables	On clamp	Off clamp	*p* value
Surgical approach, no. (%)			1
Transperitoneal	101 (82.1)	103 (81.7)	
Retroperitoneal	22 (17.9)	23 (18.3)	
Use of Airseal	13 (10.8)	9 (7.2)	0.4
CO_2_ insufflation pressure during resection (mmHg), median (IQR)	13 (12–18)	13.5 (12–18)	0.2
Resection technique (SIB score S), no. (%)			0.3
Enucleation	66 (53.7)	77 (61.1)	
Enucleoresection/resection	57 (46.3)	49 (38.9)	
Resection technique (SIB score I), no. (%)			0.3
Enucleation	64 (52)	73 (57.9)	
Enucleoresection	59 (48)	52 (41.3)	
Resection	0 (0)	1 (0.8)	
Resection technique (SIB score B), no. (%)			0.7
Enucleation	53 (43.8)	61 (48.8)	
Enucleoresection	62 (51.2)	57 (45.6)	
Resection	6 (5)	7 (5.6)	
SBP during resection (mmHg), median (IQR)	100 (90–100)	100 (90–110)	0.8
Bleeding (categorized *), no. (%)			0.2
0	23 (18.9)	21 (16.9)	
1	66 (54.1)	51 (41.1)	
2	21 (17.2)	29 (23.4)	
3	9 (7.4)	16 (12.9)	
4	2 (1.6)	6 (4.8)	
5	1 (0.8)	1 (0.8)	
Estimated blood loss (ml), median (IQR)	150 (100–200)	150 (100–250)	0.2
Renorrhaphy technique, no. (%)			0.7
Suture less	5 (4.1)	6 (4.8)	
Medullar layer only	12 (9.8)	12 (9.6)	
Cortical layer only	31 (25.2)	24 (19.2)	
Double layer	75 (61)	83 (66.4)	
Time for renorrhaphy (min), median (IQR)	10 (9–15)	10 (8–15)	0.8
Total operative time (min), median (IQR)	120 (100–180)	130 (107.5–182.5)	0.6
Use of hemostatic agents, no. (%)	113 (91.9)	108 (85.7)	0.2
Complications per patient, no. (%)			0.6
0	116 (94.3)	118 (93.6)	
1	7 (5.7)	6 (4.8)	
2	0 (0)	2 (1.6)	
Clavien grade, no. (%)			0.6
2	4 (7.5)	5 (9.8)	
3a	2 (3.8)	3 (5.9)	
3b	1 (1.9)	0 (0)	
4a	0 (0)	0 (0)	
4b	0 (0)	0 (0)	
5	0 (0)	0 (0)	
Transfusion, no. (%)	8 (6.3)	3 (3.4)	0.5
Angiography/reintervention due to bleeding, no. (%)	3 (2.7)	2 (1.7)	0.3
Positive surgical margins, no. (%)	10 (8.2)	3 (3.5)	0.1
Length of stay (d), median (IQR)	4 (3–5)	4 (3–5)	0.4
1st POD eGFR (ml/min), median (IQR)	81.3 (66.7–94.3)	85.3 (71.0–97.7)	0.2
5th POD eGFR (ml/min), median (IQR)	83.3 (70.5–93.7)	83.4 (68.6–139.3)	0.2
1st POD hemoglobin (g/dl), median (IQR)	12.4 (11.4–13.4)	12.4 (11.5–13.5)	0.7
5th POD hemoglobin (g/dl), median (IQR)	11.9 (10.8–12.8)	12.5 (11.5–13.3)	0.06
1st POD % hematocrit, median (IQR)	37.6 (35.1–40.3)	37.6 (35.0–40.4)	0.9
5th POD % hematocrit, median (IQR)	36.6 (33.0–40.0)	37.1 (34.6–40.0)	0.5

eGFR = estimated glomerular filtration rate; IQR = interquartile range; POD = postoperative day; SBP = systolic blood pressure; SIB = surface-intermediate-base.

Multivariable model analyses were performed to account for eventual unbalances. Each +1 increase in the RENAL score was found to increase protection from the occurrence of complications (OR 0.72, 95% confidence interval [CI] 0.54–0.97, *p* = 0.034). Each +1 cm increase in tumor size corresponded to an increase in the risk of blood transfusion (OR 1.39, 95% CI 1.14–1.70, *p* = 0.001). Retroperitoneal access protected from the occurrence of blood transfusions (OR 0.07, 95% CI 0.01–0.29, *p* < 0.001).

No specific impact of resection and suture techniques was found on the surgical perioperative outcomes. Finally, the higher the preoperative patient’s eGFR, the higher the probability of acute kidney injury (considered as a decrease in eGFR of >25%) measured on the 1st and 5th postoperative days (OR 1.03, 95% CI 1.01–1.06, *p* = 0.002).

## 4 Discussion

To the best of our knowledge, this is the first randomized controlled trial comparing the off- versus on-clamp approach during pure LPN. Several reports aimed to investigate the differences between the two techniques for managing the renal pedicle during the tumor resection in nephron-sparing surgery [12]. Those investigations were hindered by retrospective study design and imprecision of the outcome measurements. Systematic literature reviews pooled the limited evidence available and concluded that there is no impact of the technique used to manage the renal pedicle on either surgical or oncological outcomes [12], [19]. Only two recently published studies sought to raise the bar in the available level of evidence on this topic. Anderson and coworkers [20] performed a prospective randomized trial comparing the on- versus off-clamp technique in the setting of robotic PN. Thirty-seven versus 34 patients were analyzed. Unfortunately, the significance was not achieved in the study since the observed difference in eGFR was much less than what the authors hypothesized a priori. Our group underlined one of the issues of the study—inability to detect any variation below 10% of eGFR due to statistical matters [21]. As such, no difference in the eGFR change was reported between the groups, even if off-clamp patients had larger variability in eGFR that remained undetected. In summary, beyond the comparable perioperative outcomes, the study once again reported the “nonfinding” of no differences in renal functional outcomes irrespective of the clamping approach. Antonelli et al. [22], [23] followed the CLOCK I study. The on- and off-clamp approaches during robotic PN were confirmed to have a comparable safety profile [23]. Moreover, in the specific setting of bilateral kidneys with regular baseline function, the authors found no differences in functional outcomes [24].

We believe that our study contributes to further improving the quality of the evidence in this field. Notably, the randomization protocol worked properly, with no differences in all the preoperative baseline variables. Both clinical tumor size and tumor complexity as assessed by the RENAL score were comparable between the groups as well.

Confirming the lower level of evidence about the topic, the approaches were found comparable in the surgical perioperative outcomes, including blood loss, and complication and positive surgical margin rates.

As concerning the more advanced analysis performed to account for potential confounders, multivariable models found increasing RENAL score to be protective against the occurrence of complications (OR 0.72, 95% CI 0.54–0.97, *p* = 0.034). This could sound in contrast with literature evidence. As such, a recently published literature review confirmed the impact of higher nephrometry scores on the likelihood of having complications after PN [25]. A possible explanation is the average high experience of the surgeons who were involved in the present study. Moreover, the higher the nephrometry score, the higher the probability of being forced in performing a more enucleative technique to score with a nephron-sparing intent. A recent analysis by Minervini et al. [26] within the “Surface-Intermediate-Base consortium” found enucleation to be protective against complications if compared with the standard enucleoresection technique. Consistent with previous findings, larger tumor size increased the risk of blood transfusion (OR 1.39, 95% CI 1.14–1.70, *p* = 0.001) [5].

In our randomized study, retroperitoneal access was found to protect from blood transfusions (OR 0.07, 95% CI 0.01–0.29, *p* < 0.001), similar to the findings by Pavan et al. [27], who reported lower estimated blood loss for retroperitoneal PN in a recent systematic review and cumulative analysis of perioperative outcomes of transperitoneal versus retroperitoneal PN.

No specific impact of resection and suture techniques was found on the surgical perioperative outcomes. With the available evidence, it is hard to test the impact of such variables per se, given their interplay with other disease- and surgery-related factors [4], [5], [7], [26], [28].

Regarding the renal functional outcomes, better patient baseline renal function as assessed by eGFR predicted a higher probability of acute kidney injury on either the first or the fifth postoperative day (OR 1.03, 95% CI 1.01–1.06, *p* = 0.002). This is in line with many reports [29].

Despite the randomized controlled design, the present study is not devoid of limitations.

The most important is the fact that only expert surgeons and referral institutions participated in this study. Nevertheless, despite a rigorous randomization protocol, assigned clamping strategy was affected by subjective feelings during surgery, with the surgeon being able to shift from the off-clamp to the on-clamp approach. The likelihood of shifting from pure off-clamp to on-clamp LPN relied on tumor size and complexity. Among the cases assigned to an off-clamp procedure, 41 (32.5%) were intraoperatively converted to on-clamp LPN, but the intraoperative need to convert the planned strategy seemed harmless on the postoperative course [30]. Moreover, as per the study protocol, enrolled patients had baseline eGFR >60 ml/min, a normal contralateral kidney, and a RENAL score of ≤10. This undoubtedly does not represent the daily practice and limits the generalizability of the findings. Another bias given by unmeasurable surgeon’s skills, as well the surgeon’s attitude and experience toward a specific clamping approach should be considered. Moreover, it could be hypothesized that the peculiar setting of a surgical RCT could have prompted some additional care to preserve patient’s safety, influencing the results with respect to the daily clinical practice. Last, notwithstanding the adequate population size randomized, a small sample size of patients was analyzed relative to the number of variables collected. As such, our analysis excluded a significant relationship of clamping, resection, and renorrhaphy techniques with the outcomes of interest. However, the influence of these factors on intra- and postoperative events cannot be excluded completely, also considering the previous reports.

## 5 Conclusions

In the setting of an RCT comparing on- versus off-clamp LPN, no differences were found in the perioperative and early functional outcomes between the approaches.

  ***Author contributions*:** Riccardo Bertolo had full access to all the data in the study and takes responsibility for the integrity of the data and the accuracy of the data analysis.

*Study concept and design*: Antonelli, Cindolo, Minervini.

*Acquisition of data*: Celia, Cipriani, Falsaperla, Leonardo, Mari, Parma, Veccia, Veneziano.

*Analysis and interpretation of data*: Bertolo, Sandri.

*Drafting of the manuscript*: Bertolo.

*Critical revision of the manuscript for important intellectual content*: Bove, Antonelli, Minervini.

*Statistical analysis*: Sandri.

*Obtaining funding*: None.

*Administrative, technical, or material support*: None.

*Supervision*: Bove, Antonelli, Minervini.

*Other*: None.

  ***Financial disclosures:*** Riccardo Bertolo certifies that all conflicts of interest, including specific financial interests and relationships and affiliations relevant to the subject matter or materials discussed in the manuscript (eg, employment/affiliation, grants or funding, consultancies, honoraria, stock ownership or options, expert testimony, royalties, or patents filed, received, or pending), are the following: None.

  ***Funding/Support and role of the sponsor:*** None.
